# Complex I inhibition augments dichloroacetate cytotoxicity through enhancing oxidative stress in VM-M3 glioblastoma cells

**DOI:** 10.1371/journal.pone.0180061

**Published:** 2017-06-23

**Authors:** Nathan P. Ward, Angela M. Poff, Andrew P. Koutnik, Dominic P. D’Agostino

**Affiliations:** Department of Molecular Pharmacology & Physiology, University of South Florida, Tampa, FL, United States of America; University of Alabama at Birmingham, UNITED STATES

## Abstract

The robust glycolytic metabolism of glioblastoma multiforme (GBM) has proven them susceptible to increases in oxidative metabolism induced by the pyruvate mimetic dichloroacetate (DCA). Recent reports demonstrate that the anti-diabetic drug metformin enhances the damaging oxidative stress associated with DCA treatment in cancer cells. We sought to elucidate the role of metformin’s reported activity as a mitochondrial complex I inhibitor in the enhancement of DCA cytotoxicity in VM-M3 GBM cells. Metformin potentiated DCA-induced superoxide production, which was required for enhanced cytotoxicity towards VM-M3 cells observed with the combination. Similarly, rotenone enhanced oxidative stress resultant from DCA treatment and this too was required for the noted augmentation of cytotoxicity. Adenosine monophosphate kinase (AMPK) activation was not observed with the concentration of metformin required to enhance DCA activity. Moreover, addition of an activator of AMPK did not enhance DCA cytotoxicity, whereas an inhibitor of AMPK heightened the cytotoxicity of the combination. Our data indicate that metformin enhancement of DCA cytotoxicity is dependent on complex I inhibition. Particularly, that complex I inhibition cooperates with DCA-induction of glucose oxidation to enhance cytotoxic oxidative stress in VM-M3 GBM cells.

## Introduction

A consequence of the hallmark metabolic alterations associated with neoplastic growth is elevated oxidative stress [1]. Hypoxia, mitochondrial abnormalities, and organellar inputs, such as ER stress, not only direct cancer metabolism but also greatly influence the generation of reactive oxygen species (ROS) and oxidative stress [2, 3]. Concurrently, these energetic and redox stresses dictate a compensatory increase in antioxidant capacity that permits cancer cell resilience and proliferation [4].

ROS modulate cellular function and integrity through oxidation of macromolecular structures. Moderate oxidative stress can therefore contribute to the genomic instability that is characteristic of cancer as well as enhance oncogenic signaling through oxidation of constituents of mitogenic pathways [5]. However, excessive ROS can promote membrane dysfunction and the loss of mitochondrial integrity, ultimately leading to cell death [6].

Ionizing radiation as well as many traditional chemotherapies such as 5-fluorouracil and doxorubicin elicit cytotoxicity towards cancer cells in part through induction of ROS and overwhelming cellular redox balance [7]. Yet there is accumulating evidence that robust antioxidant capacity contributes to chemo- and radiotherapy resistance and the eventual failure of these therapies in patients [8–10]. Therefore, it is vital to identify adjuvant agents that further enhance oxidative stress to overwhelm the antioxidant system and overcome this mechanism of resistance.

The small-molecule pyruvate mimetic dichloroacetate (DCA) is being evaluated as an adjuvant to chemotherapy because of its propensity to enhance oxidative stress [11–16]. DCA, an inhibitor of pyruvate dehydrogenase kinase (PDK), promotes oxidative metabolism through activation of the pyruvate dehydrogenase complex (PDH) and subsequent flux of glucose carbon through the citric acid cycle (TCA) [17]. PDK is upregulated in a number of cancers and DCA is shown to reverse the glycolytic phenotype resultant from its enhanced activity [18].

A consequence of DCA-induced oxidative metabolism is ROS production, and this enhanced oxidative stress is shown to promote cancer cell death [19–21]. DCA potentiates the cytotoxicity of several chemotherapies and reverses HIF-mediated resistance to bevacizumab in a model of glioblastoma [11–16]. Moreover, DCA promoted stable disease in patients with malignant brain tumors in a Phase I trial [22]. However, following a separate Phase I dose-escalation study, Siu-Chung Chu et al concluded that DCA will be minimally effective as a single-agent and would be best used in combination with therapies that would benefit from enhanced oxidative metabolism [23].

There is recent evidence to suggest that DCA efficacy is enhanced by the anti-diabetic drug metformin [24, 25]. Metformin, a cationic biguanide, readily accumulates in the mitochondria, where it inhibits complex I of the electron transport chain (ETC) [26, 27]. This ETC inhibition induces energetic stress that promotes activation of adenosine monophosphate kinase (AMPK), subsequently leading to catabolic metabolism that restores energetic homeostasis [28]. Metformin is under intense investigation as an anti-cancer therapy for both tumor cell-autonomous activity as well as indirect activities in lowering systemic glucose and insulin that have largely been attributed to the reduced incidence of certain cancers in diabetic patients taking metformin [29–32].

Metformin enhanced oxidative stress and cytotoxicity in several DCA-treated breast cancer cell lines [24, 25]. DCA reversed metformin-induced glycolytic metabolism in these breast cancer cells suggesting that the enhanced oxidative stress observed with co-treatment many be resultant from oxidative glucose metabolism in the presence of metformin inhibition of complex I. Continued generation of the reducing equivalent nicotinamide adenine dinucleotide (NADH) through TCA cycling in the presence of ETC dysfunction will promote superoxide production following NADH oxidation at complex I.

We aimed to further characterize the mechanism of metformin enhancement of DCA cytotoxicity in a model of glioblastoma multiforme (GBM). Specifically, we sought to determine the necessity of complex I inhibition and AMPK activation in the reported synergy. GBMs may be particularly sensitive to a DCA and metformin combination as DCA treatment has previously exhibited some efficacy in glioma patients and metformin is shown to specifically target therapy-resistant glioblastoma stem cells (GSCs), which exhibit extraordinary antioxidant capacity [22, 33–35].

## Methods

### Cell culture

VM-M3/Fluc (VM-M3) cells were obtained as a gift from Dr. Thomas Seyfried (Boston College, Chestnut Hill, MA). They were derived from a spontaneous brain tumor in a VM/Dk inbred mouse and adapted to cell culture as previously described^36^. VM-M3 cells were cultured in D-glucose, L-glutamine, and sodium pyruvate-free Dulbecco’s Modified Eagle Medium (Gibco, Life Technologies) supplemented with 10% fetal bovine serum (Invitrogen), 25mM D-glucose (Fisher Scientific), 2mM L-glutamine (Gibco, Life Technologies), 1% penicillin-streptomycin (Invitrogen), and 10mM HEPES buffer (Gibco, Life Technologies). Cells were maintained at 37°C in 95% air, 5% CO_2_ in a humidified incubator.

### Lactate export

VM-M3 cells were seeded for 24 hours on 22-mm 12-well plates in triplicate at a density of 50,000 cells/well. The culture media was then replaced and treatment applied. To determine lactate export, 10uL of treated culture media was aspirated and applied to a lactate detection strip and lactate concentration determined with a LACTATE PLUS Lactate Meter (Nova Biomedical) at time of treatment application and every 12 hours over a period of 48 hours.

### Cell viability

Cell viability was assayed with the LIVE/DEAD Viability/Cytotoxicity Kit (Invitrogen). VM-M3 cells were seeded for 24 hours on 18-mm glass coverslips in 22-mm 12-well plates at a density of 20,000 cells/well. The culture media was then replaced and treatment applied for 24 hours. Following the 24-hour treatment, cells were washed with D-PBS (Gibco, Life Technologies) and then incubated with 800uL of 2uM Calcein AM and 4uM Ethidium Homodimer-1 (EthD-1) in D-PBS for 30 minutes. Coverslips were then inverted and mounted onto glass microscope slides and cells visualized with a Nikon TE2000E fluorescence microscope and a 10X objective lens. Calcein-AM readily passes through the membrane of intact cells and is digested by cellular esterases that yield a fluorescent calcein product (Ex/Em: 495/515 nm) that can be detected with a FITC filter as an indicator of live cells. EthD-1 (Ex/Em: 525/590 nm) is cell-impermeable but emits a red fluorescence upon association with nucleic acid following loss of membrane integrity that can be detected with a TRITC filter as an indicator of dead cells. The live/dead ratios of 10 distinct fields of view were determined via direct cell count for each treatment.

### Cytochrome c release

Apoptosis was assayed with the ApoTrack Cytochrome c Apoptosis Immunocytochemistry Kit (Abcam). 10^5^ VM-M3 cells were seeded on 22-mm glass coverslips in 35-mm 6-well plates in triplicate for 24 hours. The culture media was then replaced and treatment applied. Following treatment, cells were rinsed with D-PBS and fixed in 4% paraformaldehyde in D-PBS for 20 minutes. To improve detection signal, coverslips were then incubated in Antigen Retrieval Buffer (100mM TRIS, 5% Urea, pH 9.5) at 95°C for 10 minutes. Cells were then permeabilized in 0.1% Triton X-100 for 10 minutes and coverslips blocked in 10% goat serum for one hour. Coverslips were then incubated at 4°C overnight with mouse monoclonal antibodies for cytochrome c and mitochondrial complex Vα at a concentration of 2ug/ml. Coverslips were then rinsed in 1% goat serum and incubated in goat IgG_2a_-FITC and IgG_2b_-TXRD secondary antibodies for one hour. 10uL of DAPI mounting media was applied to a microscope slide and coverslips were inverted and mounted. Following a ten-minute incubation, fixed cells were visualized with a Nikon TE2000E fluorescence microscope and a 40X objective lens. The cytochrome c monoclonal antibody is of IgG_2a_ isotype and cytochrome c was detected with a FITC filter. The mitochondrial complex Vα monoclonal antibody is of IgG_2b_ isotype and complex Vα was detected with a Texas Red filter. Nuclear DNA was detected with a DAPI filter. Merged images were taken of 5 fields of view for each treatment.

### ROS production

Mitochondrial superoxide production was measured using the fluorescent probe, MitoSOX Red (Molecular Probes, Invitrogen). 50,000 VM-M3 cells were seeded on 18-mm glass coverslips in 22-mm 12-well plates for 24 hours. Culture media was then replaced and treatment applied. Coverslips were then rinsed with D-PBS and stained with 5uM MitoSOX Red in Hank’s Balanced Salt Solution (HBSS) with Ca^2+/^Mg^2+^ (Gibco, Life Technologies) for 10 minutes at 37°C. Coverslips were then inverted and mounted on glass microscope slides and MitoSOX Red fluorescence (Ex/Em: 510:580 nm) was detected with a TRITC filter and a Nikon TE2000E fluorescence microscope and a 40X objective lens. The average relative fluorescence intensity of individual cells within 10 fields of view were determined for each treatment.

### Mitochondrial membrane potential

Mitochondrial membrane potential (ΔΨ_m_) was measured using the cationic fluorescent probe tetramethylrhodamine (TMRE; Molecular Probes, Life Technologies). 50,000 VM-M3 cells were seeded on 18-mm glass coverslips in 22-mm 12-well plates for 24 hours. Culture media was then replaced and treatment applied. Coverslips were then rinsed with D-PBS and stained with 250nM TMRE in culture medium for 30 minutes at 37°C. Coverslips were counterstained with 100nM MitoTracker Green (Molecular Probes, Invitrogen) in culture media for 20 minutes at 37°C and then inverted and mounted on glass microscope slides. Cells were visualized with a Nikon TE2000E fluorescence microscope and a 40X objective lens. TMRE fluorescence (Ex/Em: 549/575 nm) was detected with a TRITC filter and MitoTracker Green fluorescence (Ex/Em: 490/516 nm) was detected with a FITC filter. The average relative fluorescence intensity of individual cells within 10 fields of view were determined for each treatment.

### Lipid peroxidation

Oxidative lipid damage was measured using the phenylbutadiene-based reporter for lipid peroxidation, BOPIDY® 581/591 (Molecular Probes) per the ascribed Image-iT® Lipid Peroxidation Kit protocol (Life Technologies). 50,000 VM-M3 cells were seeded on 18-mm glass coverslips in 22-mm 12-well plates for 24 hours. Culture media was then replaced and treatment applied. 10uM of the lipid peroxidation sensor was added to each well and cells incubated for 30 minutes. Cells were then washed three times with PBS and visualized with a Nikon TE2000E fluorescence microscope and a 10X objective lens. The ratio of oxidized (Ex/Em: 488/510nm, FITC filter) to reduced (Ex/Em: 580/590nm, TRITC filter) sensor fluorescence was determined for each cell in 5 distinct fields of view as an indication of the extent of lipid peroxidation induced by each treatment.

### Western blot analysis

VM-M3 cells were seeded on 35-mm 6-well plates for 24 hours at a density of 10^6^ cells/well. The culture media was then replaced and treatment applied. Cells were collected and lysed in 200uL of RIPA lysis buffer containing complete protease and phosphatase inhibitors (ThermoFisher). Lysates were centrifuged at 13,200g for 15 minutes at 4°C and the supernatant collected. Protein concentration was determined by BCA assay (ThermoFisher) and 20ug of protein was loaded into a 10% Mini-PROTEAN TGX precast polyacrylamide SDS-PAGE gel (BIO-RAD). Protein was transferred to nitrocellulose membranes, blocked with 5% non-fat dairy milk in Tris-buffered saline and tween (TBS-T) and incubated overnight at 4°C with primary antibodies for PDH-E1α (Abcam, ab110330), phospho-PDH-E1α (Ser293; Abcam, ab92696), ACC1 (Cell Signaling, #4190), and phospho-ACC (Ser79; Cell Signaling, #3661). Blots were washed with TBS-T and incubated with goat-anti-mouse and goat-anti-rabbit secondary antibodies coupled to horseradish peroxidase (HRP). HRP substrate was then applied to the blots and antibody signal was detected with the ChemiDoc MP Imaging System (BIO-RAD) or by exposure of X-ray film.

### Analysis of p-AMPKα:AMPKα

The activation status of AMPKα was assayed using the CytoGlow AMPKα (Phospho-Thr172) Colorimetric Cell-Based ELISA kit (Assay bioTech). 15,000 VM-M3 cells were seeded overnight on 96-well plates. Cells were then treated for 4-hours, washed twice with TBS, and fixed with 4% paraformaldehyde (w/v) in D-PBS) for 20 minutes. Cells were then washed 3X in Wash Buffer (0.2% Kathon CG/ICP, 1% Tween in TBS) and then incubated in Quenching Buffer (0.05% Sodium Azide, 1% H_2_O_2_ in TBS) for 20 minutes to inactivate endogenous peroxidase activity. Cells were then washed 3X with Wash Buffer and then blocked with Blocking Buffer (0.05% Sodium Azide, 0.5% Triton X-100 in TBS) for 1-hour. Cells were then incubated overnight with primary antibodies for p-AMPKα (Thr172), AMPKα or glyceraldehyde phosphate dehydrogenase (GAPDH), which served as an internal positive control. Following three washes with Wash Buffer, the cells were incubated in HRP-conjugated secondary antibodies for 90 minutes; Anti-Rabbit IgG for p-AMPK (Thr172) and AMPK, and Anti-Mouse IgG for GADPH. Cells were then washed 3X and incubated in HRP substrate (<0.02% H_2_O_2_ and < 0.1% 3,3’,5,5’-Tetramethylbenzidine [TMB]) for 30 minutes, after which 2N sulfuric acid was added to stop the peroxidase reaction. The absorbance at 450nm was then read using a plate reader (BioTek ELx800). Cells were then washed 3X and incubated with 0.05% Crystal Violet for 30 minutes. After which, the cells were washed and then incubated with SDS to solubilize the Crystal Violet for 1-hour. The absorbance was read at 595nm to quantify cell number.

### Statistical analysis

GraphPad Prism 6 software was used for all statistical analysis. Parametric tests were performed for all data sets as all groups were considered normally distributed. Paired student’s *t* tests were performed for the comparison of two groups. One-Way ANOVA with a post hoc Tukey’s multiple comparison test was performed for the comparison of more than two groups. Results were considered significant when p< 0.05.

## Results

### DCA inhibition PDH complex phosphorylation associated with enhanced ROS production

**Fig 1:**
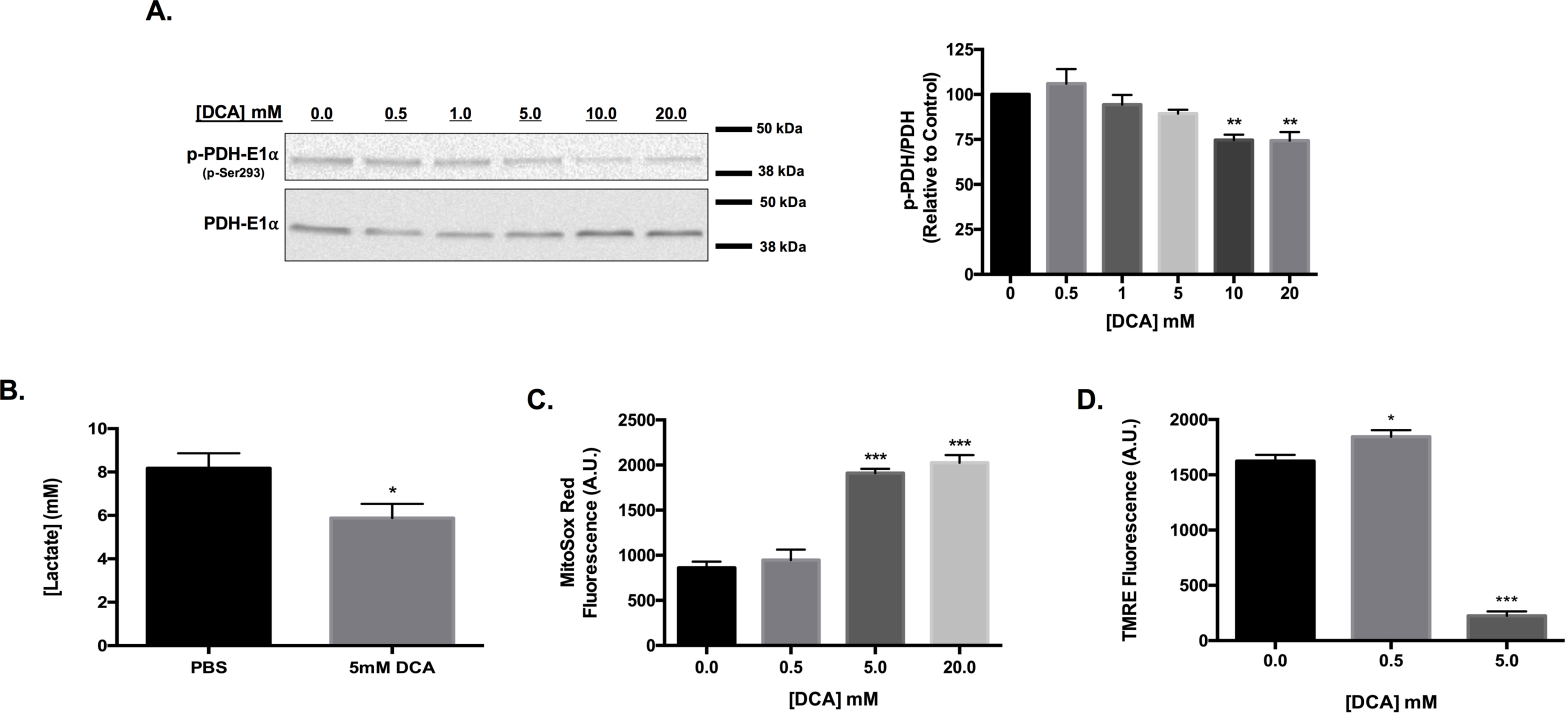
DCA promotes superoxide production and dissipation of ΔΨ_m_ in VM-M3 cells. (a) Western blot analysis of p-PDH-E1α (Ser293) and PDH-E1α in VM-M3 lysates following 4-hour treatment with DCA. Densitometric ratio of p-PDH to PDH was determined for each treatment relative to PBS control. (b) Quantification of lactate concentration in culture medium following 24-hour incubation with indicated treatment. (c) Quantification of average MitoSox Red fluorescence intensity as an indication of VM-M3 superoxide production following 1-hour incubation with DCA. (d) Quantification of average tetramethylrhodamine (TMRE) fluorescence intensity as an indication of mitochondrial membrane potential following 4-hour DCA treatment. (b) Error bars represent standard error of the mean (SEM) of three experimental replicates. (c-d) Error bars represent SEM of a single experiment replicated in triplicate; * p<0.05, and ***p<0.001.

VM-M3 cells exhibit robust basal phosphorylation of the pyruvate dehydrogenase complex (Fig 1A). DCA treatment reduced phosphorylation of the E1α subunit of the PDH complex in a concentration dependent manner following a 4-hour incubation. As PDH complex phosphorylation is associated with Warburg metabolism, we sought to determine if DCA treatment alters VM-M3 lactate production [36]. A 24-hour incubation with 5mM DCA resulted in a 28.1% reduction in lactate present in the culture medium, suggesting a shift towards glucose oxidation and away from glucose fermentation (Fig 1B).

Given that oxidative metabolism is intrinsically linked to ROS generation, we evaluated whether DCA activation of pyruvate dehydrogenase altered ROS production in VM-M3 cells. MitoSox Red fluorescent microscopy indicated a greater than two-fold increase in fluorescence intensity following 1-hour DCA treatment, indicating enhanced mitochondrial superoxide production (Fig 1C).

Changes in flux through the ETC can alter mitochondrial membrane potential, therefore we utilized tetramethylrhodamine fluorescence microscopy to determine changes in ΔΨ_m_ associated with DCA activity [37]. A 4-hour incubation with 5mM DCA resulted in significant mitochondrial depolarization, whereas treatment with a lower concentration of 500μM promoted hyperpolarization of VM-M3 mitochondria (Fig 1D). Together these results suggest that DCA-induced activation of the PDH complex promotes oxidative metabolism, which alters VM-M3 mitochondrial homeostasis.

### Modulation of oxidative stress alters DCA cytotoxicity towards VM-M3 cells

**Fig 2:**
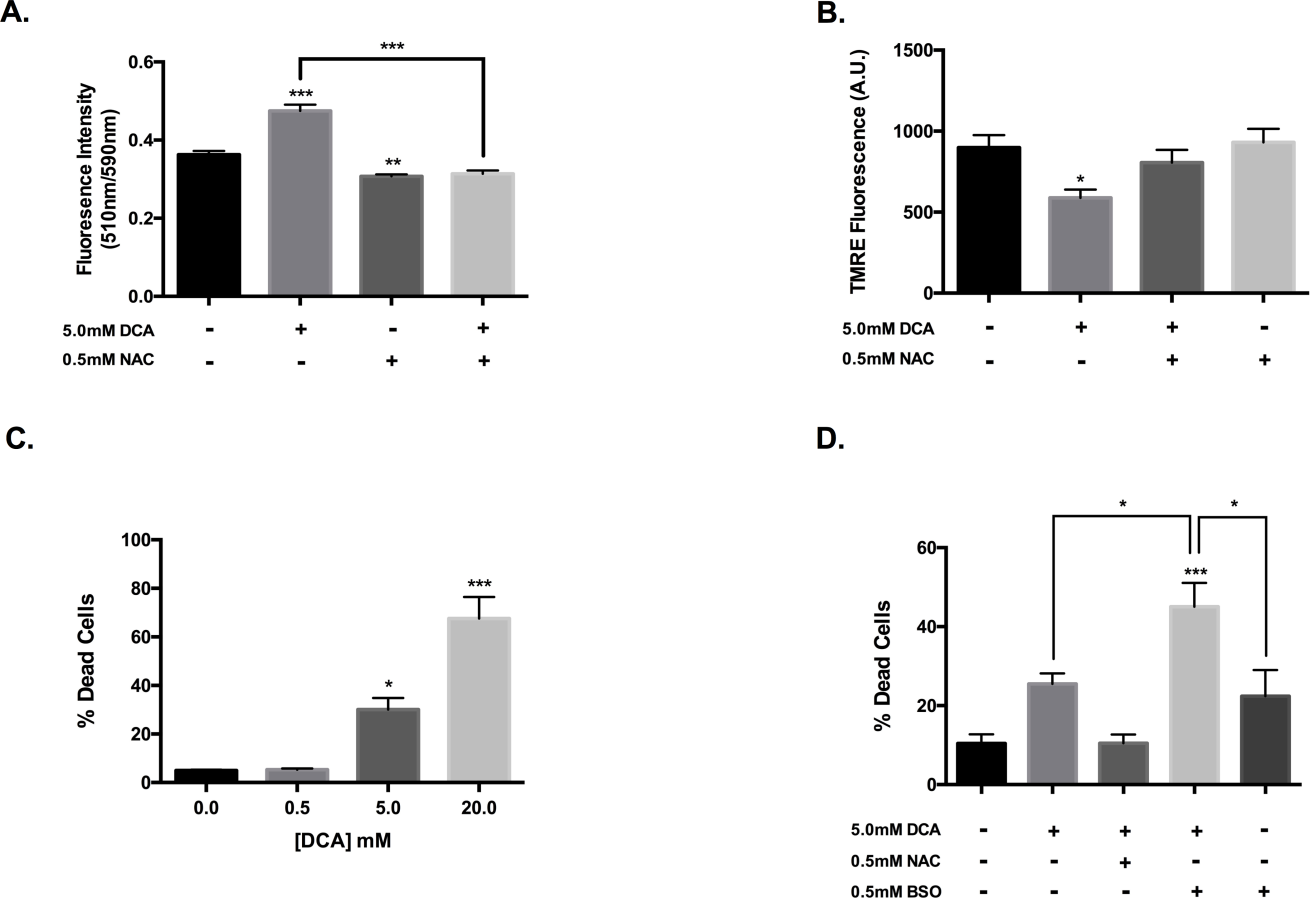
DCA cytotoxicity is dependent on oxidative stress. (a) Ratiometric detection of BOPIDY® 581/591 oxidation as an indicator of lipid peroxidation in VM-M3 cells following 4-hour treatment with DCA ± NAC. (b) Quantification of average TMRE fluorescence intensity following 4-hour DCA treatment ± N-acetylcysteine (NAC). (c) Analysis of VM-M3 viability following 24-hour treatment with DCA. Bars represent fraction of cells stained positively for ethidium homodimer-I (Ethd-1). (d) Evaluation of VM-M3 viability following 24-hour DCA treatment in the presence of modulators of glutathione availability. (a-b) Error bars represent SEM of a single experiment replicated in triplicate (c-d) Error bars represent SEM of three experimental replicates; *p<0.05, **p<0.01, and ***p<0.001.

To assess if the observed increase in mitochondrial superoxide was associated with oxidative stress, we determined the extent of lipid oxidation following DCA treatment. Indeed, lipid peroxidation was significantly increased in VM-M3 cells following 4-hour incubation with DCA (Fig 2A). This oxidative damage was attenuated with the addition of the antioxidant N-acetylcysteine (NAC). NAC co-treatment also rescued the loss of ΔΨ_m_ associated with 5mM DCA treatment (Fig 2B), suggesting the alterations to mitochondrial homeostasis are linked to oxidative stress.

To determine if this induction of oxidative stress is associated with cytotoxicity, we assayed VM-M3 viability following DCA treatment. DCA exhibits concentration-dependent cytotoxicity towards VM-M3 cells following a 24-hour incubation (Fig 2C). Treatment with 20mM DCA promotes robust cell death marked by extensive mitochondrial release of cytochrome c, indicative of apoptotic cell death (S1 Fig). Consistent with this immunofluorescent data, the addition of the pan-caspase inhibitor Z-VAD-FMK attenuated DCA cytotoxicity [38, 39] (S1 Fig).

To further establish an association between the observed increases in oxidative stress and cell death with DCA treatment, we evaluated the effects of modifying antioxidant capacity on DCA cytotoxicity. Co-incubation of 5mM DCA with the glutathione synthesis inhibitor buthionine sulfoximine (BSO) significantly enhanced cytotoxicity (Fig 2D). Conversely, addition of NAC attenuated the modest increase in cell death associated with 5mM DCA treatment. This was confirmed through immunofluorescent microscopy, which showed retention of an expansive mitochondrial network with resident cytochrome c following incubation with both DCA and NAC (S1 Fig). These data suggest that DCA-induced oxidative stress promotes loss of mitochondrial integrity and ultimately cell death in VM-M3 cells.

### Metformin enhances DCA cytotoxicity through further induction of oxidative stress

**Fig 3:**
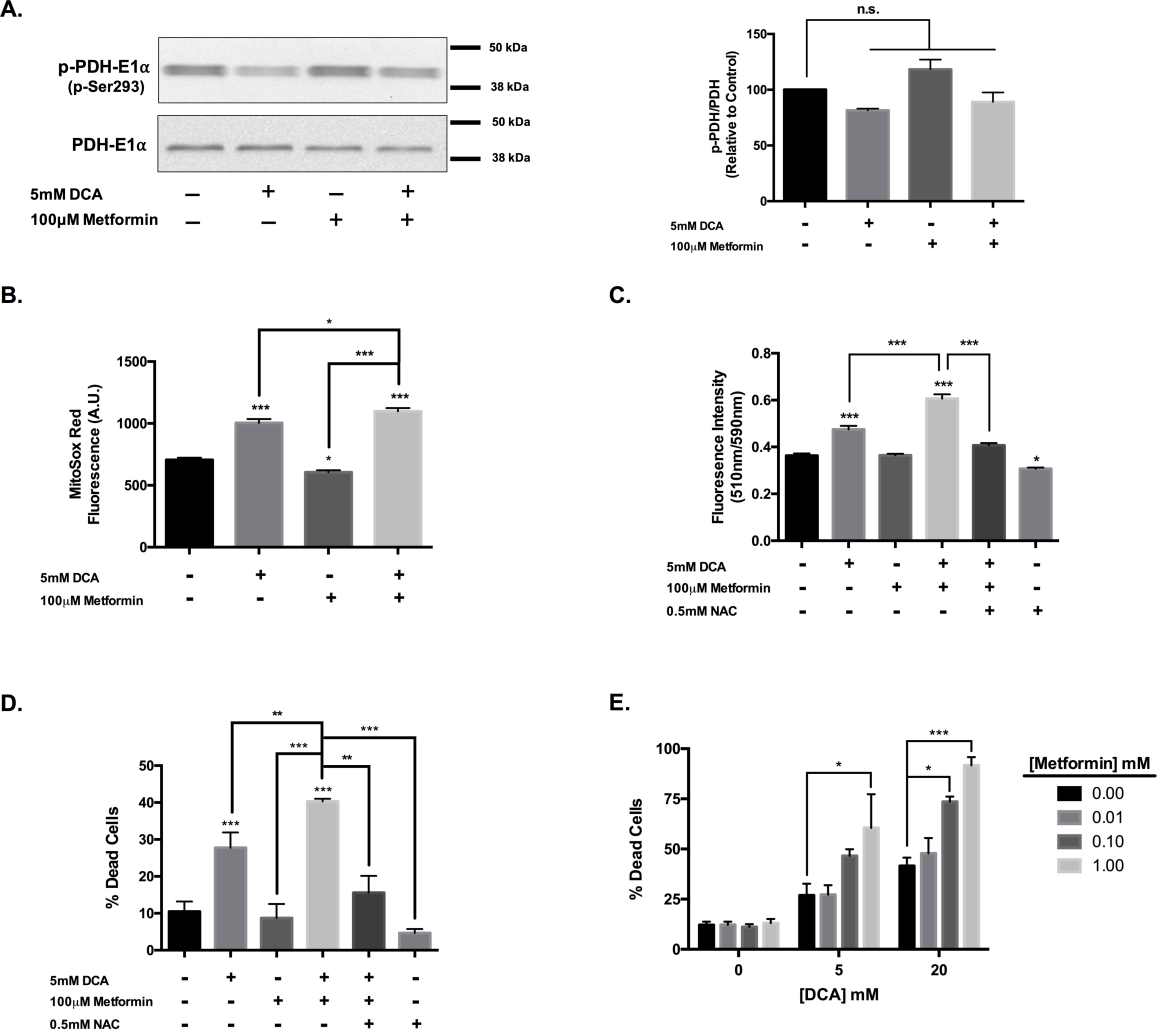
Metformin enhances DCA cytotoxicity. (a) Western blot analysis of p-PDH-E1α (Ser293) and PDH-E1α in VM-M3 cell lysates following 4-hour treatment with 5mM DCA and 100μM metformin. Densitometric ratio of p-PDH to PDH was determined for each treatment relative to PBS control. (b) Quantification of superoxide production with MitoSox Red following 1-hour treatment with DCA and metformin. (c) Quantification of BOPIDY® 581/591 oxidation as an indicator of lipid peroxidation in VM-M3 cells following 4-hour treatment with DCA and metformin ± NAC. Determination of VM-M3 cell viability following 24-hour treatment with (d) DCA and metformin ± NAC or (e) combinatorial treatment with DCA and metformin in increasing concentrations. (b, c) Error bars represent SEM of a single experiment replicated in triplicate (d, e) Error bars represent SEM of three experimental replicates; *p<0.05, **p<0.01, and ***p<0.001.

Given the observed association between induction of ROS and cytotoxicity with DCA treatment, we hypothesized that the addition of an insult to the electron transport chain would further enhance this anti-cancer activity. To assess this, we utilized the complex I inhibitor metformin. DCA promoted a modest dephosphorylation of the PDH complex (n.s., p = 0.2) even in the presence of metformin, which alone promoted a trend towards enhanced PDH phosphorylation and an associated increase in VM-M3 lactate production following a 24-hour incubation (Fig 3A, S2 Fig). Indeed, the addition of metformin further enhanced ROS production in the presence of DCA over DCA treatment alone (Fig 3B). In agreement, metformin co-treatment significantly enhanced lipid peroxidation in response to DCA treatment (Fig 3C). This oxidative damage was attenuated by NAC co-treatment.

Though metformin treatment did not elicit VM-M3 cell death (S2 Fig), the addition of 100μM metformin significantly enhanced DCA cytotoxicity following 24-hour co-treatment (Fig 3D). This effect was amplified as the concentration of either agent was increased (Fig 3E). Metformin enhancement of DCA cytotoxicity was attenuated by addition of NAC (Fig 3C). The loss of VM-M3 viability in response to combinatorial treatment was also associated with cytochrome c release and was sensitive to caspase inhibition (Fig 3D, S2 Fig). This suggests that metformin exacerbation of DCA-induced oxidative stress increased apoptotic cell death in VM-M3 cells.

### Complex I inhibition, but not AMPK activation enhances DCA cytotoxicity

**Fig 4:**
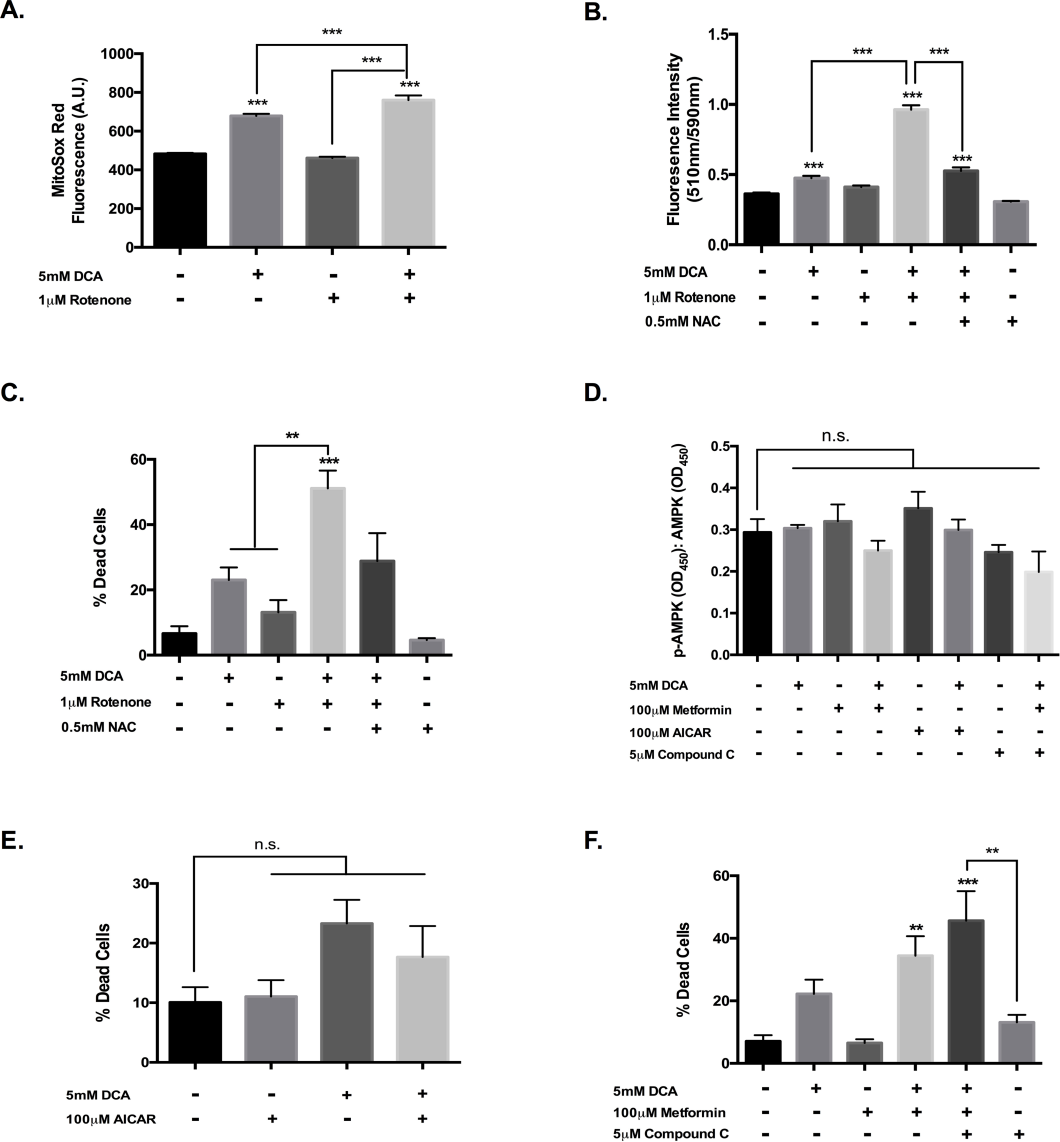
Complex I inhibition, but not AMPK activation enhances DCA cytotoxicity. (a) Average VM-M3 superoxide production following 1-hour treatment with DCA and rotenone. (b) Ratiometric detection of BOPIDY® 581/591 oxidation as an indicator of lipid peroxidation in VM-M3 cells following 4-hour treatment with DCA and rotenone ± NAC. (c-e) Analysis of rotenone, AICAR, and metformin ± compound C modulation of DCA cytotoxicity towards VM-M3 cells. (f) In-cell ELISA analysis of p-AMPKα (Thr172), and AMPKα in VM-M3 cells following 4-hour treatment with modulators of AMPK activation. (a, b, f) Error bars represent SEM of a single experiment replicated in triplicate (c-e) Error bars represent SEM of three experimental replicates; **p<0.01 and ***p<0.001.

To elucidate the contribution of complex I inhibition to metformin’s enhancement of DCA cytotoxicity, we examined the impact of rotenone, a bona fide complex I inhibitor, on DCA activity. Rotenone treatment did not affect VM-M3 superoxide production alone, but significantly enhanced the pro-oxidant effect of DCA (Fig 4A). Furthermore, the addition of rotenone significantly increased lipid peroxidation in the presence of DCA (Fig 4B). Like metformin, rotenone augmentation of DCA-induced oxidative stress was associated with enhanced cytotoxic activity that was partially attenuated by the antioxidant NAC (Fig 4C). This activity was also sensitive to caspase inhibition (S3 Fig). These results show that metformin and rotenone have a strikingly similar effect on DCA activity.

Metformin’s cellular activity is traditionally associated with AMPK activation, therefore we sought to determine if AMPK is required for metformin amplification of DCA cytotoxicity towards VM-M3 glioblastoma cells. 100μM metformin treatment did not modulate phosphorylation of AMPKα at Thr172 nor the phosphorylation state of its downstream target acetyl-CoA carboxylase 1 (ACC1) at Ser79 in VM-M3 cells (Fig 4D, S3 Fig).

5-Aminoimidazole-4-carboxamide ribonucleotide (AICAR) is an analog of 5’-AMP and is a known activator of AMPK. AICAR promoted a trend towards increased stimulatory phosphorylation of AMPK and enhanced the inhibitory phosphorylation of ACC1, which were blunted by DCA co-treatment (Fig 4D, S3 Fig). AICAR treatment did not promote VM-M3 cell death and was slightly cytoprotective in combination with DCA (Fig 4E). This was further evidenced in immunofluorescent detection of cytochrome c localization, which showed a reduction in mitochondrial stress with the combinatorial treatment (S3 Fig). Moreover, use of the AMPK inhibitor, compound c, further enhanced the efficacy of dichloroacetate and metformin in combination (Fig 4F). Collectively, these results suggest that complex I inhibition, but not AMPK stimulation is necessary for metformin enhancement of DCA cytotoxicity. In fact, AMPK activation likely diminishes the synergy between the two agents.

## Discussion

Warburg metabolism, characterized by the aerobic fermentation of glucose, is hallmark of many cancers, including GBM [4, 40]. Among the confluence of factors that contribute to this distinct metabolic phenotype is the maintenance of PDH complex phosphorylation [36]. This is mediated by enhanced PDK activity, the inhibitory kinase of the PDH complex. Pharmacological activation of this complex with the small molecule PDK inhibitor, DCA, has been shown to reduce tumor growth and promote cancer cell death through induction of oxidative stress [18, 19].

We too demonstrate that DCA activation of the PDH complex is associated with a concentration dependent increase in superoxide in VM-M3 glioblastoma cells, which show basal PDH complex phosphorylation (Fig 1A and 1C). VM-M3 cells are derived from an extremely aggressive spontaneous mouse brain tumor and exhibit a dependency on glycolytic metabolism [41, 42]. We have previously established that this tumor is susceptible to therapeutic metabolic modulation [43–45].

Changes in ETC flux drive alterations in mitochondrial membrane potential, which is derived from the movement of protons across the inner membrane upon oxidation of reducing equivalents [37]. At a low millimolar concentration, DCA promoted mitochondrial hyperpolarization, indicative of enhanced efficient electron transport [37]. Whereas increasing the concentration to a level that also stimulated ROS production resulted in dramatic membrane depolarization (Fig 1D). Abundant oxidative stress can damage membrane lipids and thus disrupt mitochondrial membrane integrity, ultimately leading to apoptotic initiation [6]. Indeed, we show that DCA treatment increased lipid peroxidation in VM-M3 cells (Fig 2A). likely contributing to the observed loss of mitochondrial homeostasis. Addition of the antioxidant NAC maintained ΔΨ_m_ and attenuated lipid peroxidation in the presence of DCA (Fig 2A and 2B), suggesting that the loss of membrane potential with high DCA (> 5mM) is associated with the robust induction of oxidative stress.

Consistent with previous reports, we show that oxidative stress in necessary for DCA cytotoxicity (Fig 2) [11–21]. Manipulating antioxidant capacity through modulation of glutathione synthesis significantly affected DCA efficacy. The γ-glutamylcysteine synthetase inhibitor, BSO, restricts production of the vital antioxidant glutathione and further augmented DCA cytotoxicity towards VM-M3 cells (Fig 2D) [46]. Conversely, providing an exogenous cysteine source for synthesis of the glutathione tripeptide in the form of NAC restored VM-M3 viability in the presence of DCA treatment (Fig 2D, S1 Fig). In line with the literature, we demonstrate a need for supraphysiological concentrations of DCA to elicit an anti-cancer effect [11–21]. As dichloroacetate exists physiologically as an anion, it is relatively membrane impermeable despite its small size and requires the mitochondrial pyruvate carrier for mitochondrial uptake [47, 48]. Pathak et al reported that conjugating DCA to a lipophilic carrier enhanced mitochondrial transport and reduced the IC_50_ value of DCA from millimolar to the low micromolar range [49]. This is well within achievable serum trough levels associated with DCA administration and reflective of the K_i_ of PDK2 (~200μM), the most ubiquitous isoform [22, 23, 47]. Suggesting that a conjugated form of DCA may elicit a more robust anti-cancer effect at physiological concentrations.

Though DCA has been shown to be an effective preclinical antineoplastic against an array of cancers, it has yielded minimal clinical benefit as a standalone therapy [11–23]. As such, much of the investigative focus on DCA has shifted towards its efficacy as an adjuvant to established therapies. DCA has been shown to be especially useful in reversing resistance to a number of agents, particularly in instances where resistance is mediated through enhanced glycolytic metabolism [13, 14, 16, 20]. Given that dichloroacetate treatment synergizes with pro-oxidant anti-cancer therapies, we hypothesized that compromising mitochondrial efficiency in the presence of DCA-induced glucose oxidation would also be synergistic.

The anti-diabetic drug metformin has displayed robust activity towards cancer, both cell-autonomous and indirect metabolic effects [50–53]. Of particular note is the observation that metformin inhibits complex I (NADH:ubiquinone oxidoreductase) of the electron transport chain [26, 27]. Inhibition of NADH oxidation and electron transfer at complex I leads to a disruption of ATP synthesis and ultimately an energetic crisis, often marked by the activation of the critical energy sensor, AMPK [54]. The activation of pyruvate dehydrogenase promotes flux of glucose carbon through the TCA cycle, subsequently increasing the rate of NADH generation [36, 47]. Thus, co-administration of DCA and metformin could potentially lead to an increase in the NADH:NAD^+^ ratio in the presence of diminished capacity to regenerate NAD^+^, precipitating a redox imbalance and oxidative stress. In fact, it has been recently reported that metformin does enhance oxidative stress in the presence of DCA treatment in breast cancer cells [24, 25]. This coincided with synergistic cytotoxicity that required the increase in oxidative stress. We sought to characterize the necessity of complex I inhibition in the efficacy of the combination.

We were able to replicate metformin potentiation of superoxide production with DCA treatment in our glioblastoma cells (Fig 3B). This corresponded with enhanced lipid peroxidation and cytotoxicity that was attenuated with NAC, further supporting the notion that metformin enhances the efficacy of dichloroacetate through exacerbation of oxidative stress (Fig 3C and 3D). The traditional mitochondrial poison rotenone elicited remarkably similar effects on VM-M3 cells in combination with DCA. Rotenone inhibits the transfer of electrons from iron sulfur clusters resident in complex I of the ETC to ubiquinone, leading to inefficient NADH oxidation [55]. Unlike metformin, which decreased VM-M3 ROS production, rotenone treatment did not affect superoxide generation (Fig 4A). This suggests differing mechanisms of complex I inhibition; the mechanism of metformin action at complex I is not fully understood. Nonetheless, rotenone also potentiated oxidative stress upon co-incubation with DCA (Fig 4A and 4B). Rotenone had a greater effect on VM-M3 viability in combination with a modestly cytotoxic concentration of DCA than metformin (Fig 4C). This is likely an effect of the degree of complex I inhibition as metformin is thought to be only a mild inhibitor of complex I [54]. The structurally related biguanide, phenformin, is considered more potent than metformin in part because of enhanced lipophilicity that facilitates increased mitochondrial uptake. However, where metformin is well tolerated clinically, phenformin has been removed from the clinic over concerns of lactic acidosis [56].

Metformin treatment is often associated with the stimulation of AMPK, which contributes to the anti-diabetic activity of the agent [57]. The concentration of metformin required to enhance DCA anti-cancer activity towards VM-M3 cells did not promote AMPK activation or modulation of the downstream target ACC1, suggesting AMPK activity is not necessary for the observed cytotoxicity (Fig 4D, S3 Fig). Indeed, AICAR did not further augment DCA-induced cell death (Fig 4E). Rather, AICAR co-treatment was protective, preventing the mitochondrial stress seen with DCA treatment (S3 Fig). This is consistent with AMPK’s role as an energy sensor and survival mediator. Moreover, compound c antagonization of AMPK further enhanced the cytotoxicity of DCA and metformin (Fig 4F). This suggests that the combination may be most effective in the absence of AMPK, such as in LKB1-deficent tumors [58]. LKB1 is responsible for the stimulatory phosphorylation of AMPK in response to energetic stress [59].

These data suggest that complex I inhibition cooperates with DCA activation of oxidative glucose metabolism to promote catastrophic oxidative stress in VM-M3 glioblastoma cells. There is extraordinary interest in targeting cancer mitochondria as a therapeutic strategy as recent evidence suggests mitochondrial metabolism is required for tumorigenesis and to meet the bioenergetics demands or rapidly proliferating tumor cells [26, 60, 61]. As mitochondrial metabolism is intrinsically linked to redox balance, a known sensitivity of cancer, targeting the organelle is likely to prove successful [7]. Schöckel et al recently reported that inhibition of complex I with an experimental small molecule induced cytotoxic oxidative stress and inhibited tumor growth in a model of melanoma, a highly aggressive tumor species [62]. Our results also demonstrate efficacy in targeting the efficiency of electron transport in an aggressive cancer, as GBM is a highly malignant brain tumor associated with an extremely poor prognosis [63].

Experimental GBMs have previously shown sensitivity to DCA modulation of glucose metabolism [14, 64]. Moreover, Shen et al showed efficacy in the dual-targeting of GBM metabolism with DCA and a mitochondrial poison [65]. Metformin may be particularly useful in combination with DCA in GBMs because of an observed sensitivity of GSCs to metformin [33–35]. Cancer stem cells are a fractional cell subpopulation within tumors that are believed to contribute to chemoresistance and eventually recurrent disease. Evidence suggests that this inherent resistance is a result of immense antioxidant capacity [8–10]. Thus, metformin may sensitize this critical cell population to the pro-oxidant effect of a DCA and metformin combination, perhaps through disruption of glutathione synthesis [66]. Indeed, recently Jiang et al reported that DCA enhanced the efficacy of phenformin in prolonging survival in an orthotopic GSC model [67].

## Conclusions

Our results provide further evidence for potential synergy between DCA and metformin in targeting GBM. Specifically, that modulation of redox balance through insult of mitochondrial metabolic efficiency is a potential anti-cancer strategy that merits further evaluation. This combination may be particularly useful as an adjuvant to current pro-oxidant therapies, for which efficacy is often fleeting due to chemoresistant mechanisms that restrict mitochondrial oxidation [13, 14, 16, 20].

## Supporting information

S1 Fig DCA treatment promotes apoptotic cell death. (a) Representative merged immunofluorescent images of VM-M3 cells following 12-hour treatment with DCA ± NAC. Fixed cells were stained for cytochrome c (green) and mitochondrial complex Vα (red) and counterstained with DAPI (blue). Scale bars represent 50μm. Pearson’s correlation coefficient determined for each cell within 5 fields of view. (b) Analysis of VM-M3 viability following 24-hour DCA treatment ± the pan-caspase inhibitor Z-VAD-FMK. Error bars represent SEM of three experimental replicates; **p<0.01 and ***p<0.001.
(TIFF)

S2 Fig Metformin treatment does not diminish VM-M3 viability. (a) Determination of the lactate concentration in culture medium following 24-hour incubation with vehicle or metformin. (b) Analysis of VM-M3 viability following 24-hour treatment with a range of metformin concentrations. (c) Representative merged immunofluorescent images of cytochrome c localization in VM-M3 cells following 12-hour treatment with DCA and metformin. Scale bars represent 50μm. Pearson’s correlation coefficient determined for each cell within 5 fields of view. (d) Analysis of VM-M3 viability following 24-hour DCA and metformin treatment ± Z-VAD-FMK. (a, b, d) Error bars represent SEM of three experimental replicates; *p<0.05 and **p<0.01.
(TIFF)

S3 Fig AICAR protects VM-M3 cells from DCA-induced stress. (a) Quantification of VM-M3 cell death following DCA and rotenone treatment ± Z-VAD-FMK. (b) Western blot analysis of p-ACC (Ser79) and ACC1 in VM-M3 cell lysates following 4-hour treatment with 5mM DCA and 100μM metformin or 100μM AICAR. Densitometric ratio of p-ACC to ACC was determined for each treatment relative to PBS control. (c) Representative merged immunofluorescent images of cytochrome c localization in VM-M3 cells following 12-hour treatment with DCA and AICAR. Scale bars represent 50μm. Pearson’s correlation coefficient determined for each cell within 5 fields of view. (a, b) Error bars represent SEM of three experimental replicates; *p<0.05 and **p<0.01.
(TIFF)

S1 File Data tables. (PDF)

S2 File Raw images. (XLSX)
