# Supplementary figures and images for: Complex I inhibition augments dichloroacetate cytotoxicity through enhancing oxidative stress in VM-M3 glioblastoma cells
Source: PLoS One. 2017 Jun 23;12(6):e0180061. doi: 10.1371/journal.pone.0180061 (PMC5482478; doi:10.1371/journal.pone.0180061)

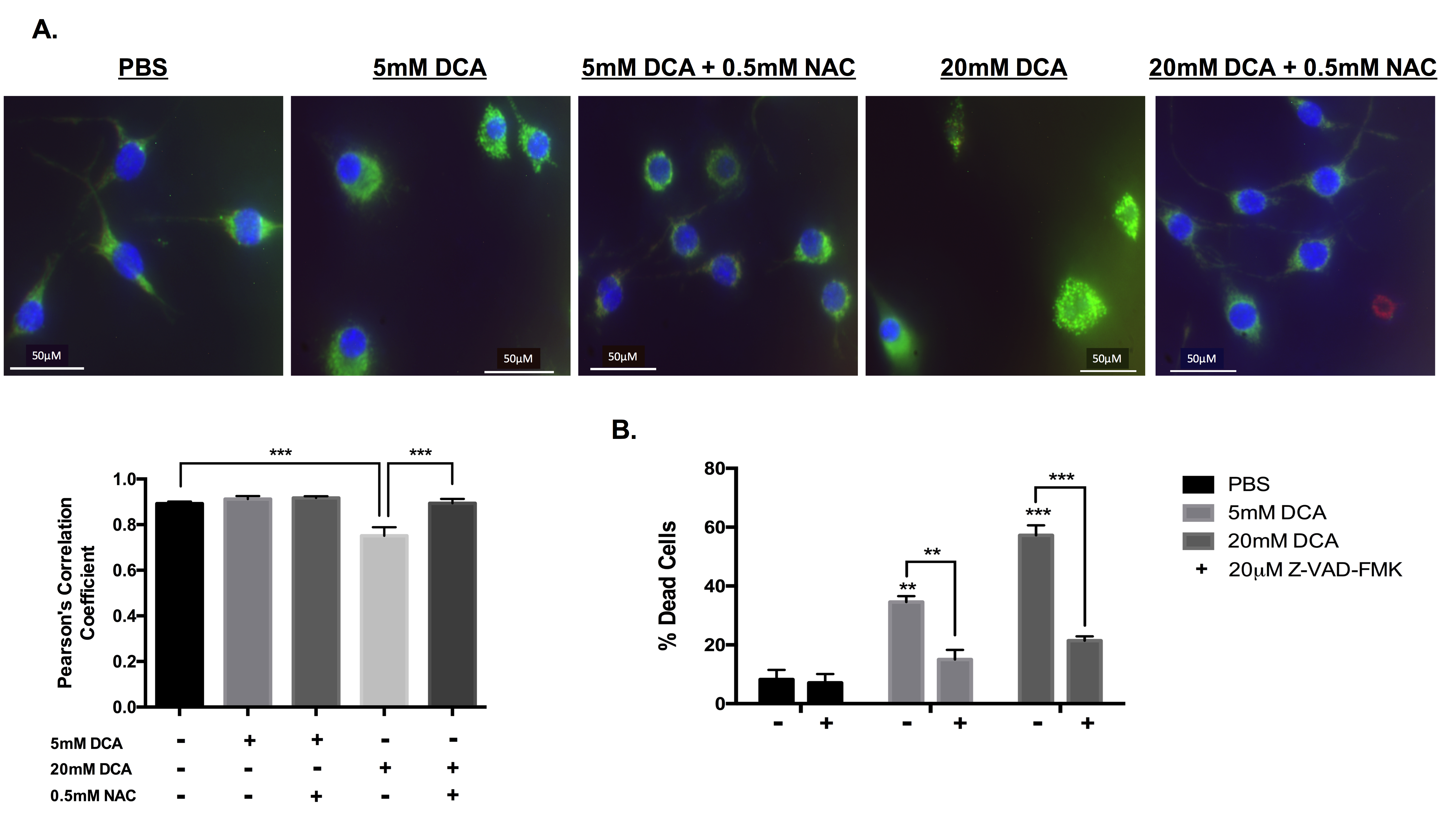

Supplement: S1 Fig — (a) Representative merged immunofluorescent images of VM-M3 cells following 12-hour treatment with DCA ± NAC. Fixed cells were stained for cytochrome c (green) and mitochondrial complex Vα (red) and counterstained with DAPI (blue). Scale bars represent 50μm. Pearson’s correlation coefficient determined for each cell within 5 fields of view. (b) Analysis of VM-M3 viability following 24-hour DCA treatment ± the pan-caspase inhibitor Z-VAD-FMK. Error bars represent SEM of three experimental replicates; **p<0.01 and ***p<0.001. (TIFF) [file pone.0180061.s001.tiff]

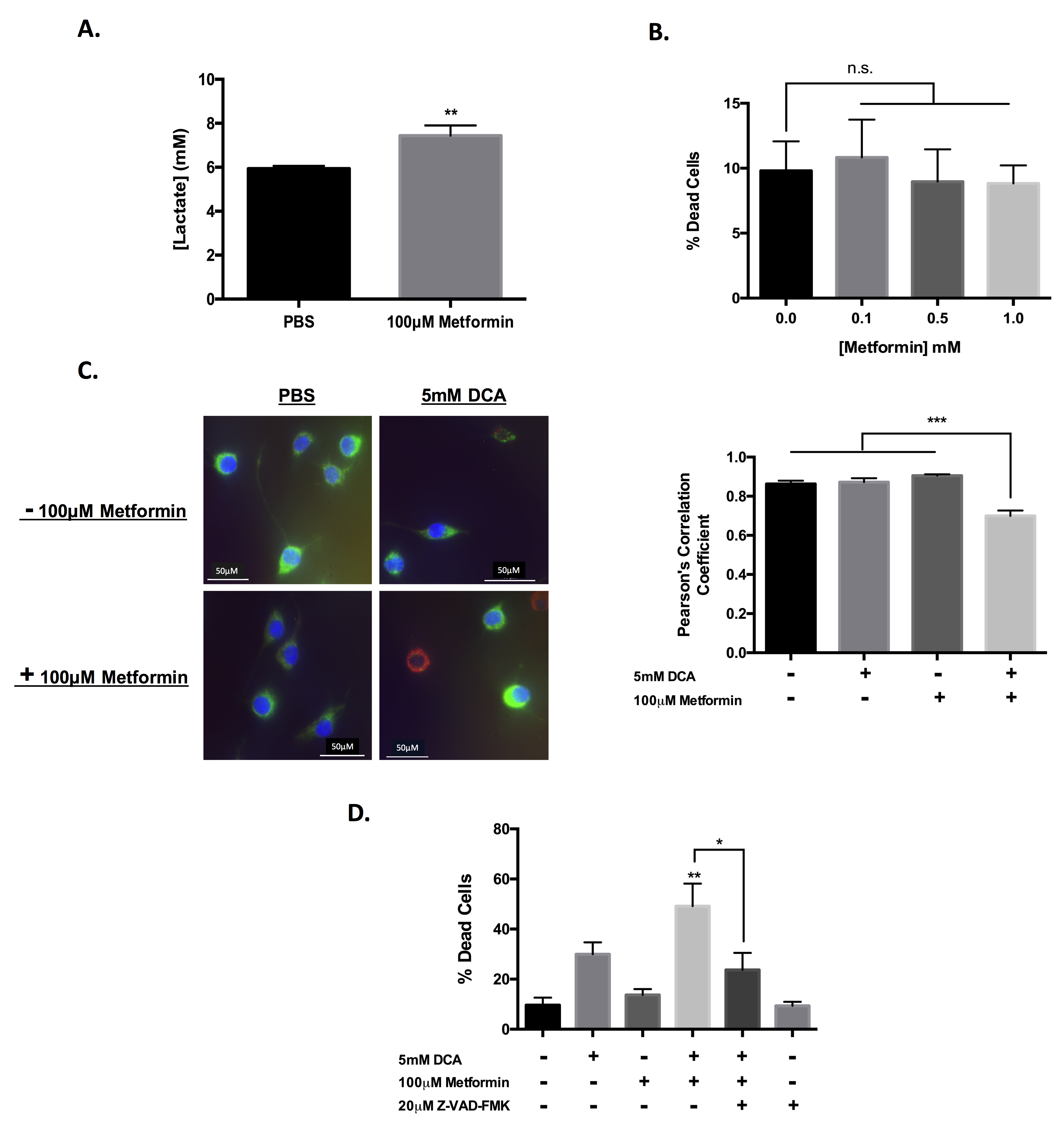

Supplement: S2 Fig — (a) Determination of the lactate concentration in culture medium following 24-hour incubation with vehicle or metformin. (b) Analysis of VM-M3 viability following 24-hour treatment with a range of metformin concentrations. (c) Representative merged immunofluorescent images of cytochrome c localization in VM-M3 cells following 12-hour treatment with DCA and metformin. Scale bars represent 50μm. Pearson’s correlation coefficient determined for each cell within 5 fields of view. (d) Analysis of VM-M3 viability following 24-hour DCA and metformin treatment ± Z-VAD-FMK. (a, b, d) Error bars represent SEM of three experimental replicates; *p<0.05 and **p<0.01. (TIFF) [file pone.0180061.s002.tiff]

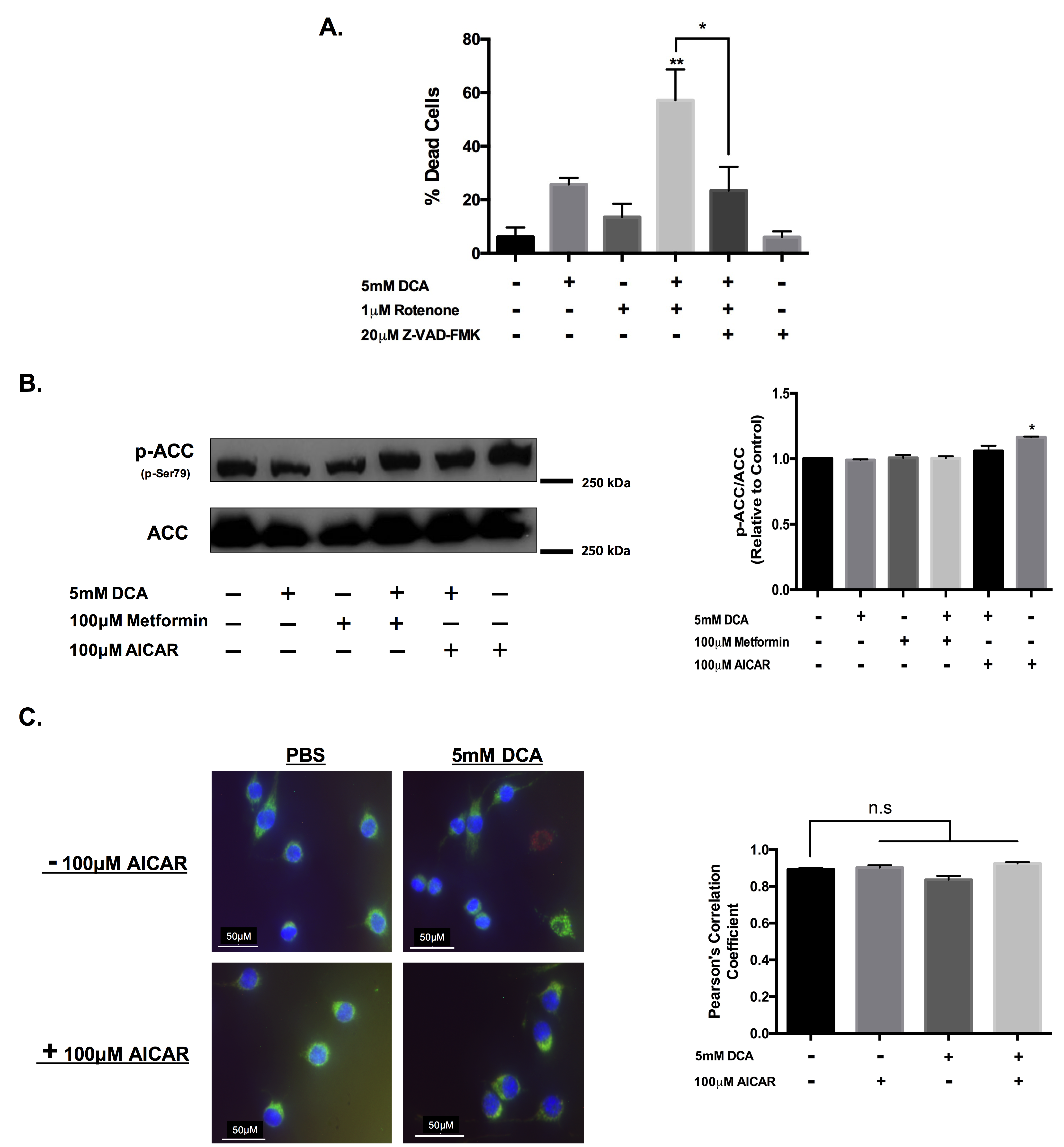

Supplement: S3 Fig — (a) Quantification of VM-M3 cell death following DCA and rotenone treatment ± Z-VAD-FMK. (b) Western blot analysis of p-ACC (Ser79) and ACC1 in VM-M3 cell lysates following 4-hour treatment with 5mM DCA and 100μM metformin or 100μM AICAR. Densitometric ratio of p-ACC to ACC was determined for each treatment relative to PBS control. (c) Representative merged immunofluorescent images of cytochrome c localization in VM-M3 cells following 12-hour treatment with DCA and AICAR. Scale bars represent 50μm. Pearson’s correlation coefficient determined for each cell within 5 fields of view. (a, b) Error bars represent SEM of three experimental replicates; *p<0.05 and **p<0.01. (TIFF) [file pone.0180061.s003.tiff]
