# Supplementary material for: Complex I inhibition augments dichloroacetate cytotoxicity through enhancing oxidative stress in VM-M3 glioblastoma cells
Source: PLoS One. 2017 Jun 23;12(6):e0180061. doi: 10.1371/journal.pone.0180061 (PMC5482478; doi:10.1371/journal.pone.0180061)

Fig 1A

p-PDH-E1α

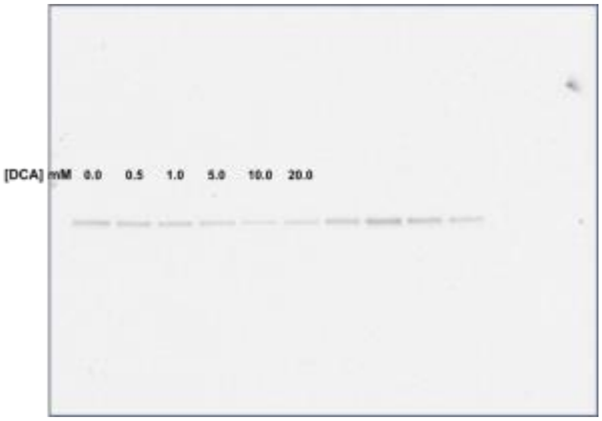

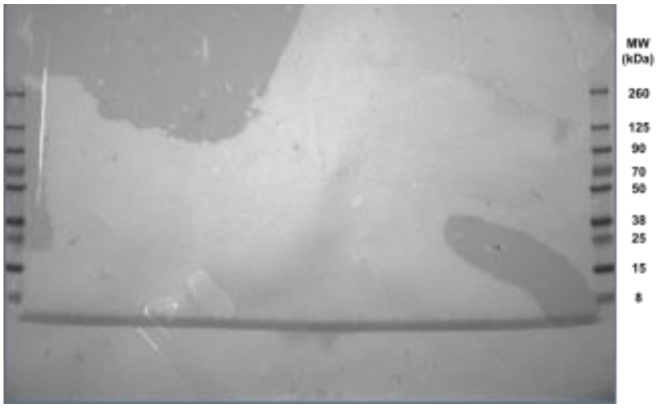

PDH- E1α

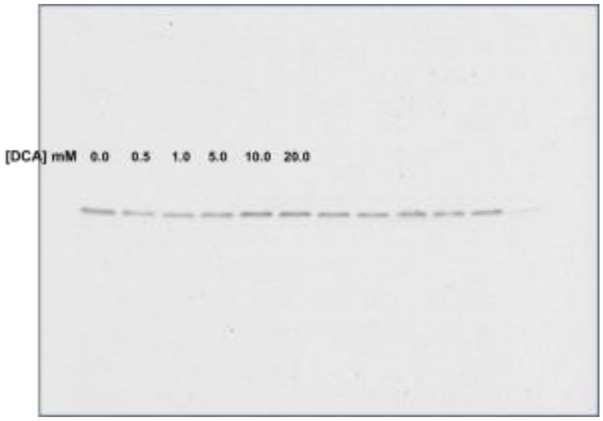

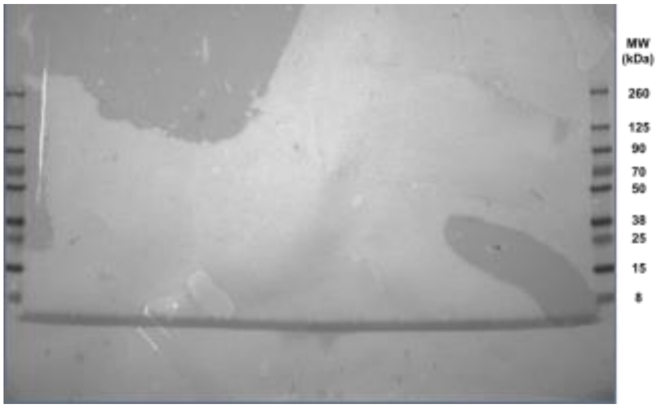

Fig 3A

p-PDH-E1α

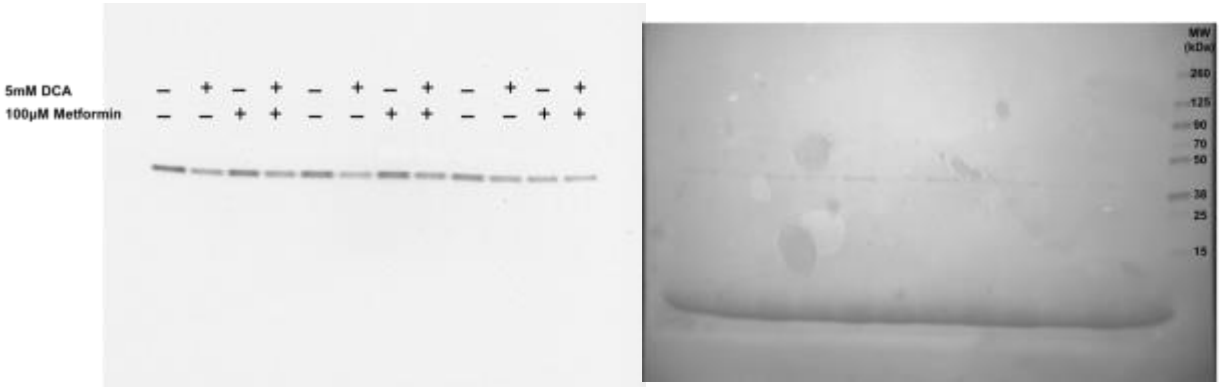

PDH-E1α

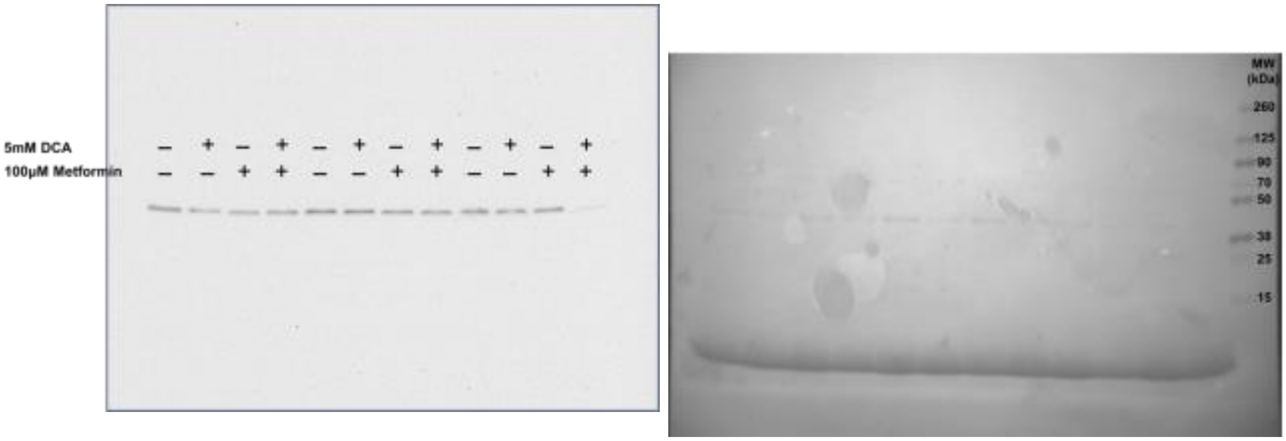

S1 Fig A

PBS

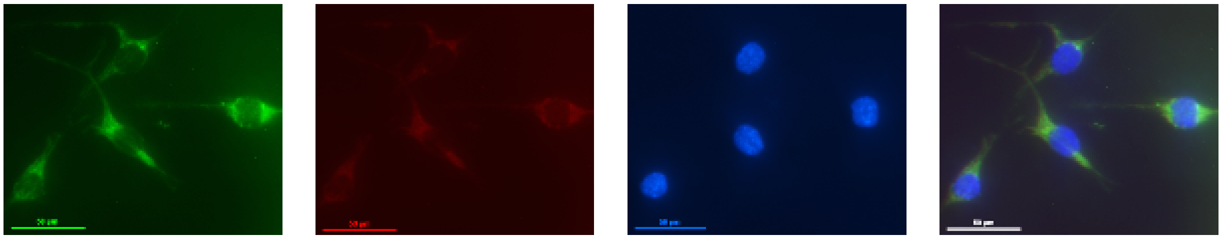

5mM DCA

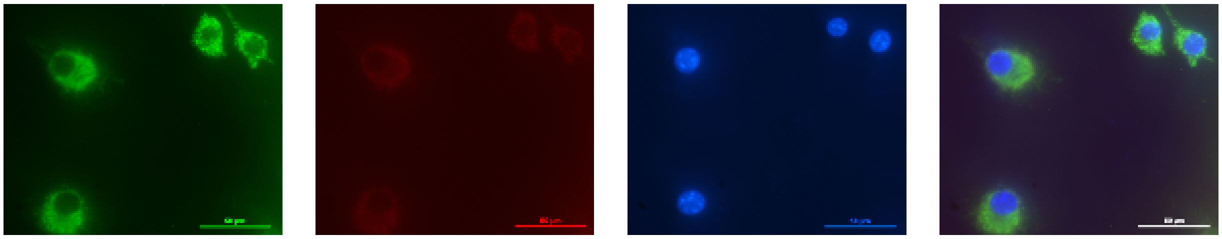

5mM DCA + 0.5mM NAC

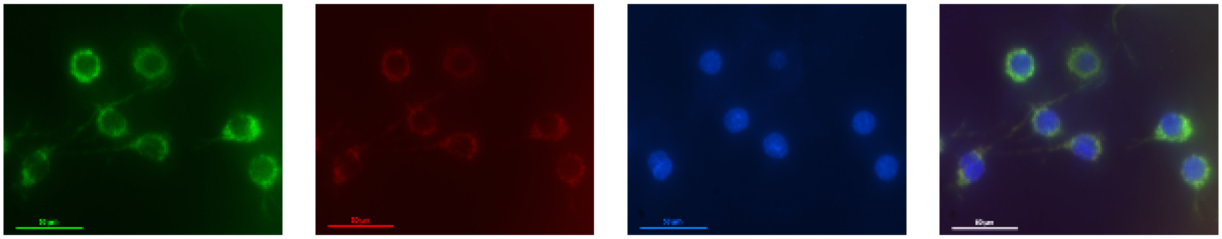

20mM DCA

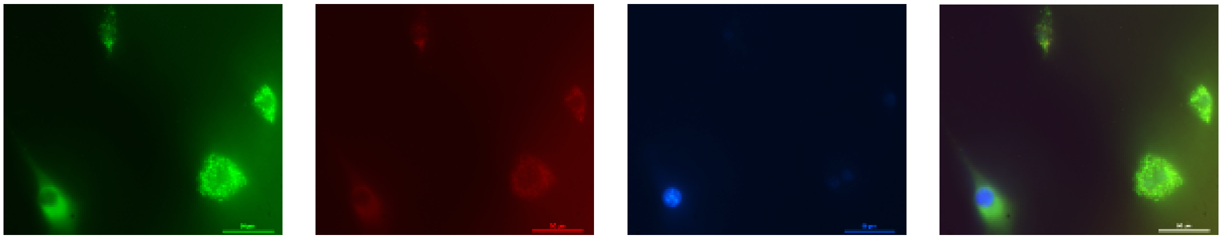

20mM DCA + 0.5mM NAC

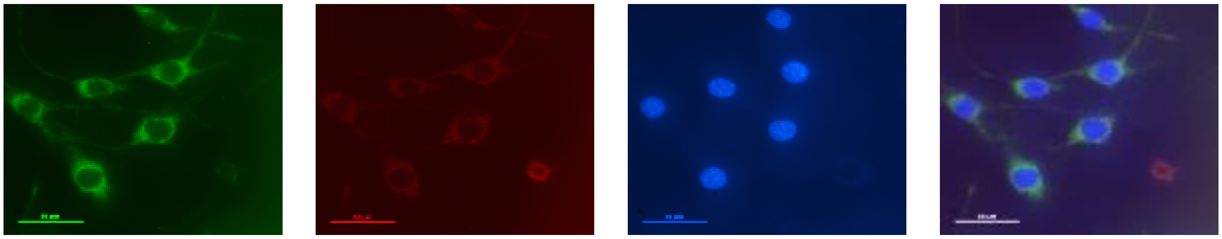

S2 Fig C

PBS

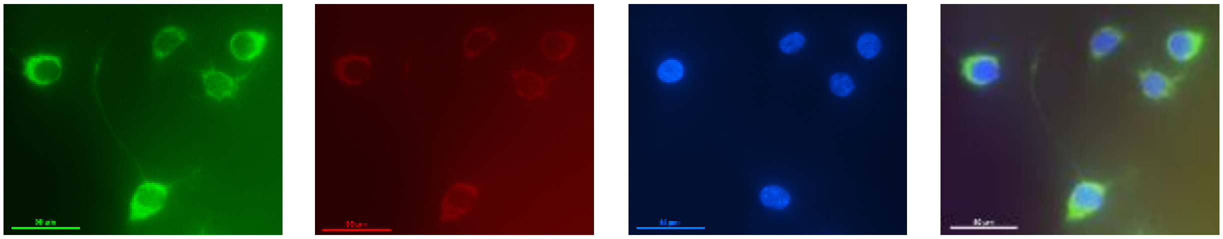

5mM DCA

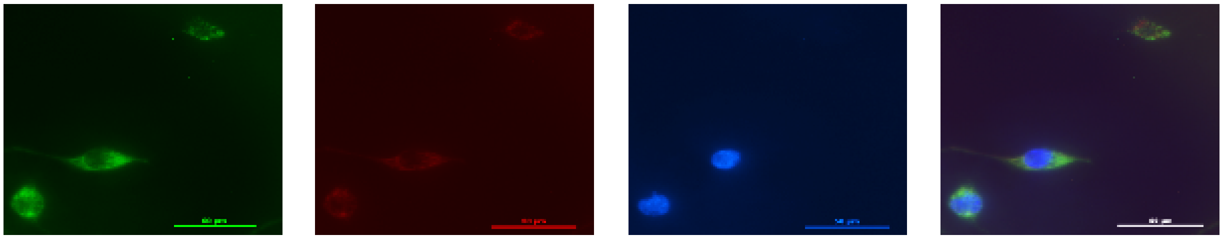

100µM Metformin

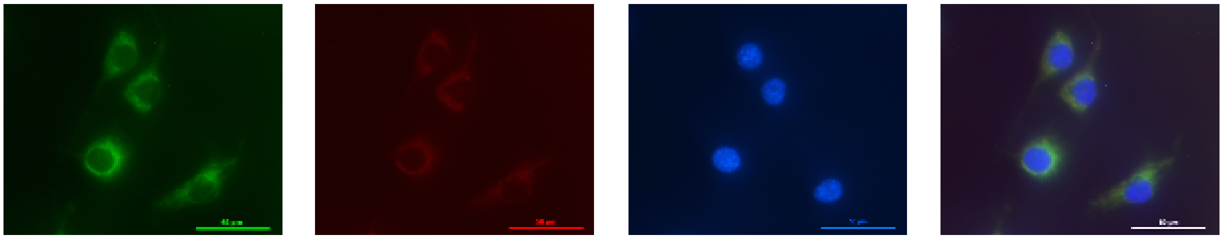

5mM DCA + 100µM Metformin

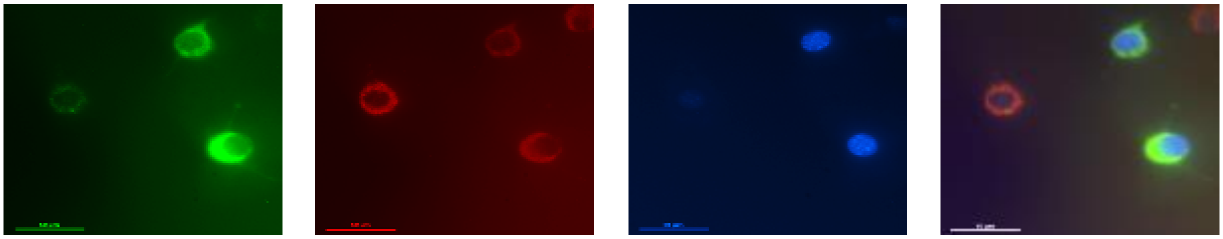

<u>S3 Fig B</u>

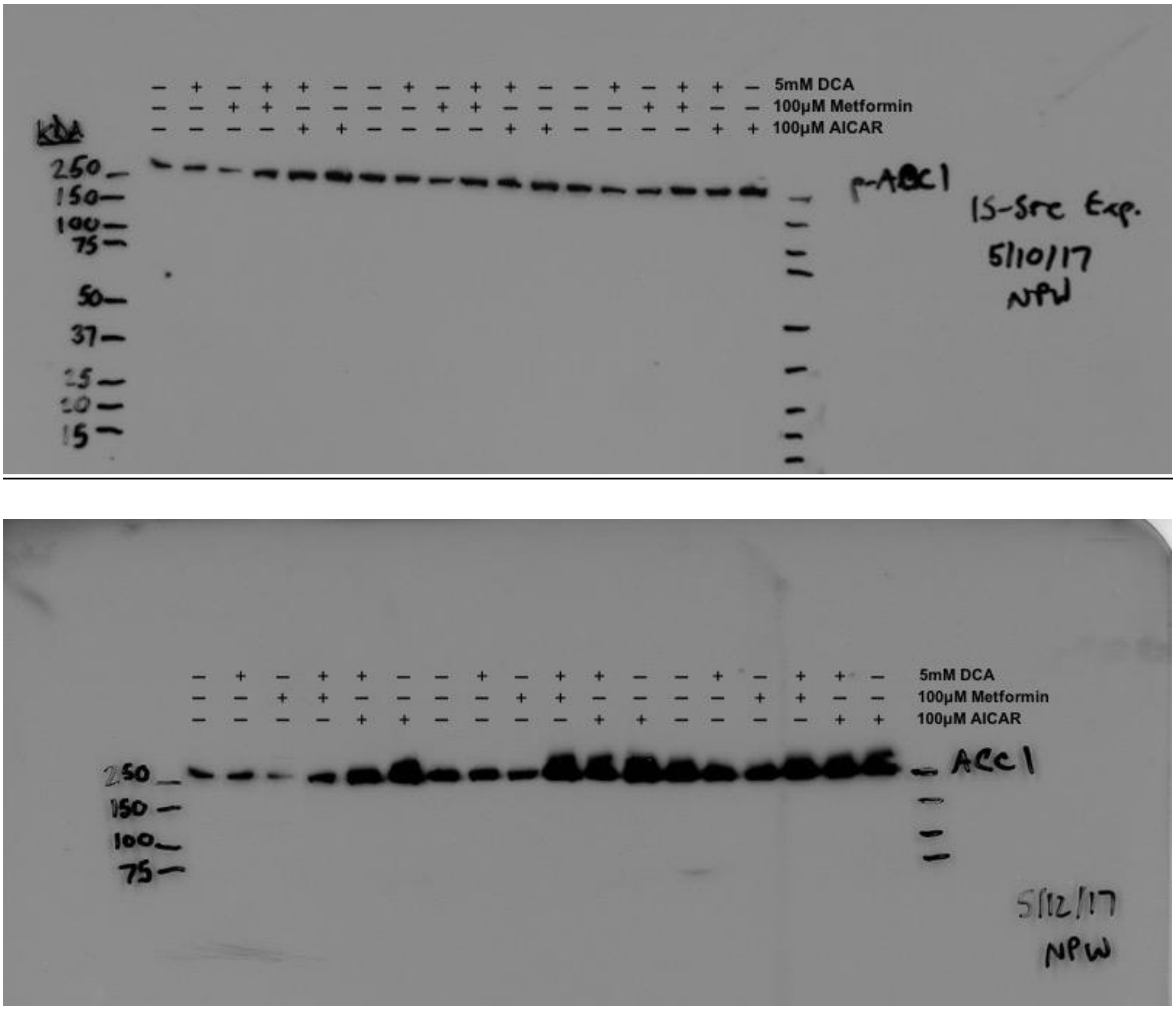

S3 Fig C

PBS

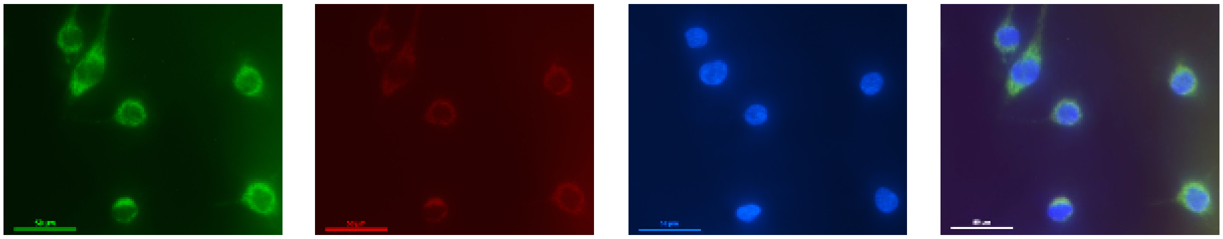

5mM DCA

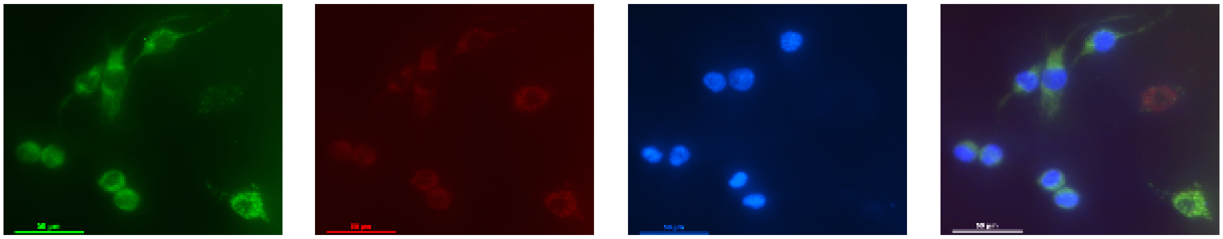

100µM AICAR

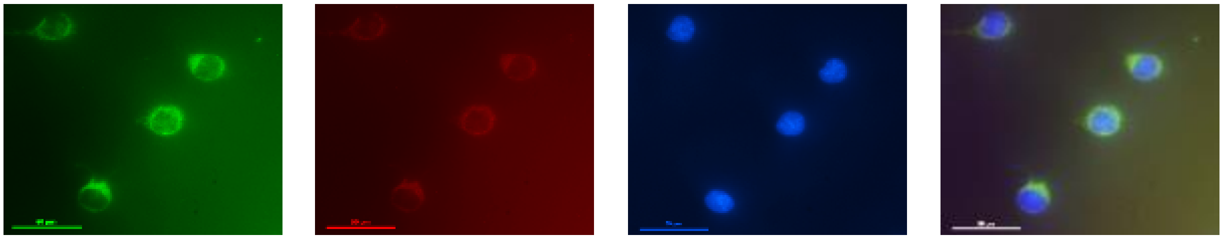

5mM DCA + 100µM AICAR

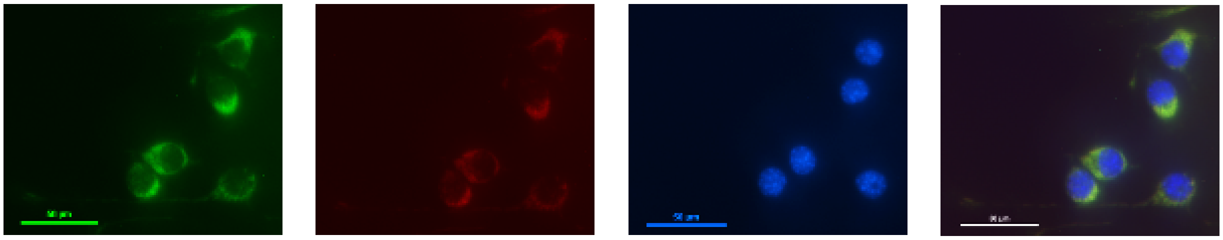

Supplement: S1 File — (PDF) [file pone.0180061.s004.pdf]
